# A Multi-Head Attention Network for Fast Prediction of Ultrasonic Guided Wave Dispersion Under Coupled Temperature and Stress

**DOI:** 10.3390/s26051549

**Published:** 2026-03-01

**Authors:** Xiao Ying, Zhao Wang, Jian Li, Yantao Liu, Haibo Li, Haoran Jin, Fuzai Lv, Yang Liu

**Affiliations:** 1 State Key Laboratory of Precision Measuring Technology and Instruments, Tianjin University, Tianjin 300072, China; yingxx@tju.edu.cn (X.Y.); 3015205286@tju.edu.cn (Z.W.); tjupipe@tju.edu.cn (J.L.); 2 Beijing Institute of Structure and Environment Engineering, Beijing 100076, China; lytus@126.com (Y.L.); haibo_lihb@aliyun.com (H.L.); 3 State Key Laboratory of Fluid Power and Mechatronic Systems, Zhejiang University, Hangzhou 310027, China; jinhr@zju.edu.cn (H.J.); lfzlfz@zju.edu.cn (F.L.)

**Keywords:** extreme environment, ultrasonic guided waves, dispersion curves, deep learning

## Abstract

**Highlights:**

**What are the main findings?**

- A Multi-Head Attention Network (D-MHAN) is proposed to accurately map eleven physical parameters to guided wave dispersion curves under coupled temperature–stress conditions, achieving a Pearson correlation coefficient of 0.9999.
- The deep learning model achieves computational speeds approximately 30 and 168 times faster than the Semi-Analytical Finite Element (SAFE) and Wave Finite Element Method (WFEM) approaches, respectively.

**What are the implications of the main findings?**

- The millisecond-level prediction capability overcomes the computational bottleneck of traditional methods, enabling real-time environmental effect compensation and calibration for ultrasonic sensors.
- The quantified parameter sensitivity analysis provides a theoretical basis for selecting optimal sensor wave modes to enhance monitoring robustness in extreme environments.

**Abstract:**

Ultrasonic guided wave sensors are widely employed for structural health monitoring; yet, their signal interpretation reliability is frequently compromised in extreme environments where coupled temperature and stress induce significant nonlinear drifts in dispersion characteristics. To overcome the computational bottleneck of conventional numerical methods that hinders real-time sensor calibration, this paper proposes a Dispersion Multi-Head Attention Network (D-MHAN) that directly maps eleven raw physical parameters to full-band dispersion responses. By adopting a non-normalized input strategy to internalize underlying physical laws, the model enables robust out-of-distribution extrapolation even when material properties exceed the training manifold. It was validated against a high-fidelity dataset spanning temperatures from −250 °C to 100 °C and stresses from 0 to 150 MPa generated by the Semi-Analytical Finite Element (SAFE) method. The proposed D-MHAN achieves a Pearson correlation coefficient of 0.9999 and provides computational speeds approximately 30 and 168 times faster than SAFE and the Wave Finite Element Method (WFEM). The model’s practical utility is further corroborated by cryogenic experiments on an aerospace storage tank. This work establishes a critical foundation for real-time parameter sensitivity analysis and environmental effect compensation in practical ultrasonic sensing applications.

## 1. Introduction

Major national equipment—such as aerospace vehicles, deep-sea exploration platforms, and advanced energy systems—must maintain safe operation under extreme temperatures and complex coupled loads. Achieving the required high reliability throughout the service life presents both a core scientific challenge and a significant engineering bottleneck [1,2,3]. For instance, ensuring the structural stability and reliability of aerospace cryogenic fuel tanks under extreme cryogenic temperatures reaching −250 °C and high stresses up to 150 MPa induced by internal pressure has become a critical technology for achieving spacecraft reusability. Therefore, developing advanced structural health monitoring (SHM) technologies for the real-time, accurate prediction of damage evolution and performance degradation in key load-bearing structures holds critical strategic significance [4,5]. Among various diagnostic approaches, ultrasonic guided wave technology has emerged as a prominent route for both SHM and non-destructive evaluation. Its unique advantages include wide-area coverage from a single excitation, high sensitivity to early micro-defects, and ease of system deployment [6,7].

The propagation behavior of guided waves, particularly their dispersion characteristics, encapsulates rich information about the structural configuration and environmental state, forming the theoretical basis for damage localization, stress assessment, and material state characterization. However, dispersion varies strongly with material, geometry, and environment [8,9]. This sensitivity benefits monitoring but complicates signal interpretation and remains a major obstacle to reliable application under complex conditions.

Under extreme environments, the coupled influence of temperature and stress exerts a particularly pronounced and highly nonlinear effect on guided wave dispersion, which causes the collected sensor signals to deviate significantly from the reference baseline, leading to frequent false alarms and compromising the reliability of the sensing system. To accurately capture this behavior, extensive research efforts have been devoted worldwide from both theoretical and experimental perspectives [10,11,12,13]. Regarding temperature effects, early investigations primarily focused on empirical measurements and phenomenological observations. Seale et al. experimentally examined the temperature-induced drift of Lamb wave dispersion curves in composite plates [12]. Roy et al. established a numerical model incorporating the temperature dependence of material properties to predict and compensate for guided wave signal variations [13]. With the deepening of such studies, researchers have shifted toward developing more universal theoretical models. Among them, the baseline signal stretching and resampling method proposed by Michaels has become one of the classical algorithms for temperature compensation [14,15].

Regarding stress effects, most existing studies are grounded in the framework of acoustoelastic theory [16]. The early foundations of this theory were established by Hughes and Kelly [17], after which Gandhi et al. derived the dispersion equations of acoustoelastic Lamb waves in prestressed plates based on the same theoretical formulation [18]. To account for the stress-induced effects in structures with complex geometries, researchers have progressively integrated analytical models with numerical techniques [19,20,21]. Loveday et al. [20] combined the acoustoelastic theory with the Semi-Analytical Finite Element (SAFE) method and developed the Acoustoelastic–SAFE (AE-SAFE) model, which efficiently analyzes guided wave propagation in waveguides of arbitrary cross-section under axial stress. Yang et al. [21] employed the Rotated Staggered-Grid Finite-Difference (RSG-FD) method to solve the acoustoelastic wave equations incorporating third-order elastic constants, simulating elastic wave propagation in prestressed media under multiple loading conditions, including hydrostatic, uniaxial, pure shear, and simple shear states. Their results revealed the velocity anisotropy and the corresponding influence of prestress on wavefront morphology and amplitude.

In recent years, the research focus has gradually shifted toward the more complex temperature–stress coupling problem. In such scenarios, temperature-dependent variations in elastic properties and stress-induced acoustoelastic effects act simultaneously, often leading to pronounced dispersion drift. Liu et al. experimentally quantified the temperature dependence of ultrasonic longitudinal guided waves in long-range steel strands and observed measurable drifts in wave speed and time of flight, providing direct evidence to support thermo-mechanical compensation in stress evaluation [22]. For railway structures, Zhao et al. analyzed guided-wave dispersion characteristics of rails under thermal stress and discussed mode selection, highlighting the necessity of dispersion-aware modeling and mode choice in thermally stressed rails [23]. Guided-wave methods have also been applied to in situ assessment of rail thermal stress/neutral temperature in continuous welded rails, where an implementable inversion procedure was established through combined theoretical, numerical, and experimental validation [23]. In addition, thermo-acoustoelastic Lamb-wave studies and semi-analytical modeling frameworks further support systematic dispersion evaluation under coupled thermo-mechanical conditions [24].

Although the above physics-based approaches are theoretically rigorous, their computational processes are highly time-consuming. Particularly when evaluating coupled temperature and stress effects over wide parametric ranges, it is necessary to independently solve the dispersion curves for every individual working condition within a vast multidimensional parameter space. Such point-by-point computations cause the total computational cost to grow exponentially, making it difficult to satisfy the embedded processing constraints of intelligent sensors. Consequently, this computational bottleneck severely limits the capability of ultrasonic sensors to perform real-time self-calibration and signal compensation under extreme environments [25,26].

In recent years, the rapid advancement of artificial intelligence has provided a new paradigm for overcoming this challenge. Within the field of guided wave inspection, AI-driven methods have been widely adopted for signal processing, damage detection and localization, as well as high-precision tomographic reconstruction, achieving remarkable success [27,28,29,30]. However, the application of AI to forward prediction of the physical propagation behavior of guided waves—especially the dispersion characteristics under multi-physics coupling conditions—remains insufficiently explored [31]. Yang et al. employed a Fully Connected Neural Network (FCNN) to achieve fast prediction of guided wave dispersion curves in pipelines [32]. Nevertheless, its relatively simple architecture limits its capability to learn the complex nonlinear mappings between input parameters and dispersion responses, making it difficult to provide precise physical priors required for high-fidelity sensor signal interpretation under multi-field coupling.

To address this limitation, we developed a Multi-Head Attention Network (MHAN)-driven deep learning approach for the fast and accurate prediction of guided wave dispersion under extreme temperature–stress coupling conditions. The model ingests eleven key physical parameters to capture these intricate interactions: the material’s initial density (*ρ*_0_), Young’s modulus (*E*_0_), and Poisson’s ratio (*ν*_0_); the third-order Murnaghan constants (*l*, *m*, *n*); the linear thermal expansion coefficient ($\alpha_{L}$); the temperature coefficients for Young’s modulus and Poisson’s ratio ($K_{E}$, $K_{\nu }$); and the operational temperature (*T*) and stress (σ). This enables an end-to-end mapping from environmental conditions to the full-band dispersion response of the sensor. A large-scale, high-fidelity dispersion database is constructed for model training and validation. The proposed framework is systematically evaluated in terms of its prediction accuracy across multimodal and broadband regimes, as well as its generalization capability beyond the training domain. The results demonstrate that the method provides an efficient and accurate solution for ultrasonic sensors, enabling real-time dispersion calibration, online parameter sensitivity analysis, and dynamic environmental effect compensation under extreme environments.

## 2. Materials and Methods

### 2.1. Theory

#### 2.1.1. Fundamentals of Ultrasonic Guided Wave Dispersion

Ultrasonic guided waves are elastic waves that can propagate over long distances within solid structures. Their propagation behavior is jointly governed by the geometric configuration, material properties, and environmental conditions of the structure. In isotropic elastic media, guided waves vary as plane waves along the propagation direction, while their distribution across the cross-section is determined by the combined effects of the boundary conditions and the governing wave equations. The guided-wave displacement field is expressed as follows:

(1) $$u(x,y,z,t)=u(y,z)e^{i(kx\;-\omega t)}$$

where u(y,z) describes the displacement distribution across the cross-section, *k* represents the wavenumber along the propagation direction, and ω denotes the angular frequency.

For an infinite plate, the displacement field is decomposed into longitudinal and transverse potentials. The corresponding longitudinal and transverse wave velocities are determined by the Lamé constants *λ* and *μ* and the material density *ρ* as follows:

(2) $$\left\{\begin{array}{ccc} c_{L} & = & \sqrt{\frac{\lambda \;+2\mu }{\rho }}\\ c_{T} & = & \sqrt{\frac{\mu }{\rho }} \end{array}\right.$$

For a plate of thickness 2 *h*, the symmetric (S) and antisymmetric (A) Lamb-wave dispersion relations are [6]:

(3) $$\left\{\begin{array}{c} \frac{tan(qh)}{tan(ph)}=-\frac{4k^{2}pq}{({k^{2}-q}^{2}{)}^{2}},S\;Mode\\ \frac{tan(qh)}{tan(ph)}=-\frac{(q^{2}-k^{2}{)}^{2}}{4k^{2}pq},A\;Mode \end{array}\right.$$

where

(4) $$p^{2}=\frac{\omega^{2}}{c_{L}^{2}}-{\;k}^{2}$$

(5) $$q^{2}=\frac{\omega^{2}}{c_{T}^{2}}-k^{2}$$

For a given ω, solving the dispersion equation yields the wavenumber, from which phase and group velocities are derived as:

(6) $$C_{p}=\frac{\omega }{k}$$

(7) $$C_{g}=\frac{\partial \omega }{\partial k}$$

These relations form the fundamental theoretical framework describing the dispersion behavior of ultrasonic guided waves, providing the physical basis for subsequent analysis of wave propagation within coupled thermal-stress environments.

#### 2.1.2. Mechanism of Temperature–Stress Coupling on Guided Wave Dispersion

The dispersion characteristics of guided waves comprehensively reflect the coupled influence of structural geometry and material constitutive behavior on wave propagation. In isotropic solids, density, Young’s modulus, and Poisson’s ratio determine the second-order Lamé constants *λ* and *μ*, which, in turn, govern the morphology of dispersion curves and the distribution of wave modes. Under multi-physical coupling involving temperature and stress, variations in these material parameters induce additional complexity in the dispersion behavior.

The theoretical framework of this study is grounded in acoustoelastic theory and the Murnaghan constitutive model to characterize the dynamic elastic response under multi-physics coupling. To focus on the nonlinear mapping between external fields and dispersion, the material is assumed to remain within the linear elastic regime, thereby excluding residual stress and phase transitions from the current formulation. Under these assumptions, the second-order Lamé constants under the reference temperature $T_{0}$ and zero external stress is expressed as:

(8) $$\lambda_{0}=\frac{E_{0}\nu_{0}}{\left(1+\nu_{0})(1-2\nu_{0}\right)}$$

(9) $$\mu_{0}=\frac{E_{0}}{2(1+\nu_{0})}$$

Temperature variation changes the Lamé constants through thermal softening and expansion:

(10) $$\lambda_{T}=\frac{E(T)\nu (T)}{\left[1+\nu (T)][1-2\nu (T)\right]}$$

(11) $$\mu_{T}=\frac{E(T)}{2[1+\nu (T)]}$$

where E(T) and ν(T) are temperature-dependent Young’s modulus and Poisson’s ratio, expressed linearly as

(12) $$E(T)={\;E}_{0}[1-K_{E}\Delta T]$$

(13) $$\nu (T)=\nu_{0}[1+K_{\nu }\Delta T]$$

where *E*_0_ and *ν*_0_ represent the values of Young’s modulus and Poisson’s ratio at the reference temperature, while *K_E_* and *K_ν_* are the corresponding temperature coefficients.

The material density is also affected by thermal expansion and can be modified as

(14) $$\rho (T)=\frac{\rho_{0}}{1+3\alpha_{L}\Delta T}$$

where $\rho_{0}$ is the density at the reference temperature and zero stress, and *α_L_* is the linear thermal expansion coefficient.

Under an externally applied static stress *σ*, the mechanical response of the material to a small dynamic perturbation is governed by its effective tangent stiffness tensor. For an initially isotropic material subjected to a uniform stress *σ* and temperature *T*, the effective tangent stiffness can be represented as the sum of the temperature-dependent initial stiffness and a stress-related correction term [17]:

(15) $$C_{ijkl}=C_{ijkl}^{\left(2\right)}(\lambda ,\mu ,T)+C_{ijkl}^{\left(3\right)}(l,m,n,\sigma )$$

(16) $$C_{ijkl}^{\left(2\right)}(\lambda ,\mu ,T)=\lambda_{T}\delta_{ij}\delta_{kl}+\mu_{T}(\delta_{ik}\delta_{jl}+\delta_{il}\delta_{jk})$$

where *l*, *m*, and *n* are the Murnaghan constants, which account for the third-order contributions to the stiffness and explicitly depend on the stress state. When the principal axis of uniaxial stress aligns with the propagation direction, the acoustoelastic correction can be equivalently expressed as a modification to the second-order Lamé constants:

(17) $$\lambda_{T,\sigma \;}=\lambda_{T}+\Delta \lambda (\sigma )$$

(18) $$\mu_{T,\sigma }={\;\mu }_{T}+\Delta \mu (\sigma )$$

where Δλ(σ) and Δμ(σ) are linear combinations of the third-order elastic constants and the applied stress.

The corresponding longitudinal and transverse wave velocities after the acoustoelastic modification are then given by

(19) $$\left\{\begin{array}{c} c_{L}(T,\sigma )=\sqrt{\frac{\lambda_{T,\sigma }+2\mu_{T,\sigma }}{\rho (T)}}\\ c_{T}(T,\sigma )=\sqrt{\frac{\mu_{T,\sigma }}{\rho (T)}} \end{array}\right.$$

The combined influence of temperature and stress alters both *λ* and *μ*, thereby modifying the root distribution of the dispersion equations. Consequently, the guided wave dispersion curves exhibit a nonlinear coupling effect between the thermal and mechanical fields. This coupling mechanism not only governs the evolution of guided wave propagation characteristics under extreme environments but also provides the physical foundation for developing data-driven deep learning models aimed at fast and accurate dispersion prediction.

### 2.2. Methodology

#### 2.2.1. Numerical Modeling and Dataset Construction

A high-fidelity dispersion database spanning a wide parameter space is essential for training the deep-learning framework. The SAFE method was adopted for its balance between computational efficiency and numerical accuracy. The temperature–stress coupled guided-wave theory was integrated into the SAFE model to generate large-scale training data.

In the SAFE framework [33,34], guided waves are assumed to propagate as a plane wave along the axial direction, while the displacement distribution over the cross-section is discretized using finite elements. To focus the investigation on the nonlinear coupling effects of temperature and stress, this study adopts Lamb waves—the most widely employed modes in plate-like structures—and utilizes isotropic plates with a uniform thickness as the primary research object. This simplified model ensures that the observed dispersion variations are primarily driven by the material-level thermo-mechanical interactions. The established framework and the D-MHAN predictive process can be subsequently extended to waveguides with more complex cross-sections and geometric features.

To incorporate the aforementioned multi-physics coupling effects into this framework, the constitutive relation is expressed in terms of the effective tangent stiffness matrix as

(20) $$\sigma_{\Delta }=C_{eff}\varepsilon$$

where $\sigma_{\Delta }$ represents the incremental stress vector induced by small dynamic perturbations of the ultrasonic guided wave, superimposed on the initial static stress field of the material. ***ε*** denotes the infinitesimal strain tensor associated with the dynamic deformation caused by wave propagation. ***C****_eff_* defines the effective tangent stiffness matrix, characterizing the dynamic elastic response of the material under given temperature *T* and static stress *σ*. It incorporates the combined thermo-mechanical effects and depends on *T*, *σ*, and the Murnaghan constants *l*, *m*, and *n*. Accordingly, an effective stiffness matrix capable of accurately describing the temperature–stress coupling effect is obtained through the joint application of temperature and stress corrections. In this process, the material parameters are first modulated by the thermal field, and these temperature-dependent properties then serve as the baseline for calculating the stress-induced acoustoelastic corrections, thereby ensuring the physical coupling of the two fields.

By substituting the constitutive relation in Equation (20) into the weak form of the elastodynamic equation and applying finite element discretization over the waveguide cross-section Ω, following the standard SAFE procedure [33,34], the following quadratic eigenvalue problem with respect to the wavenumber *k* can be formulated:

(21) $$[K_{0}+ikK_{1}-k^{2}K_{2}]d\;=\omega^{2}M(T)d$$

where $K_{0},\;K_{1},\;K_{2}$ denote the stiffness matrix, which embeds the temperature- and stress-dependent elastic properties; ***M***(*T*) is the temperature-dependent mass matrix; and ***d*** is the nodal displacement eigenvector. For each prescribed ω, solving the eigenvalue equation yields a set of complex wavenumbers, from which the real part is used to compute the corresponding phase velocity $C_{p}$.

To construct a comprehensive and high-accuracy dispersion database spanning a broad parameter space, the physical quantities listed in Table 1 are adopted as input variables. Randomized dense sampling is performed within the predefined parameter ranges to ensure sufficient coverage across material, geometric, and environmental conditions. The representative intervals are determined based on typical engineering materials, as summarized in Table 1.

**Figure 1:**
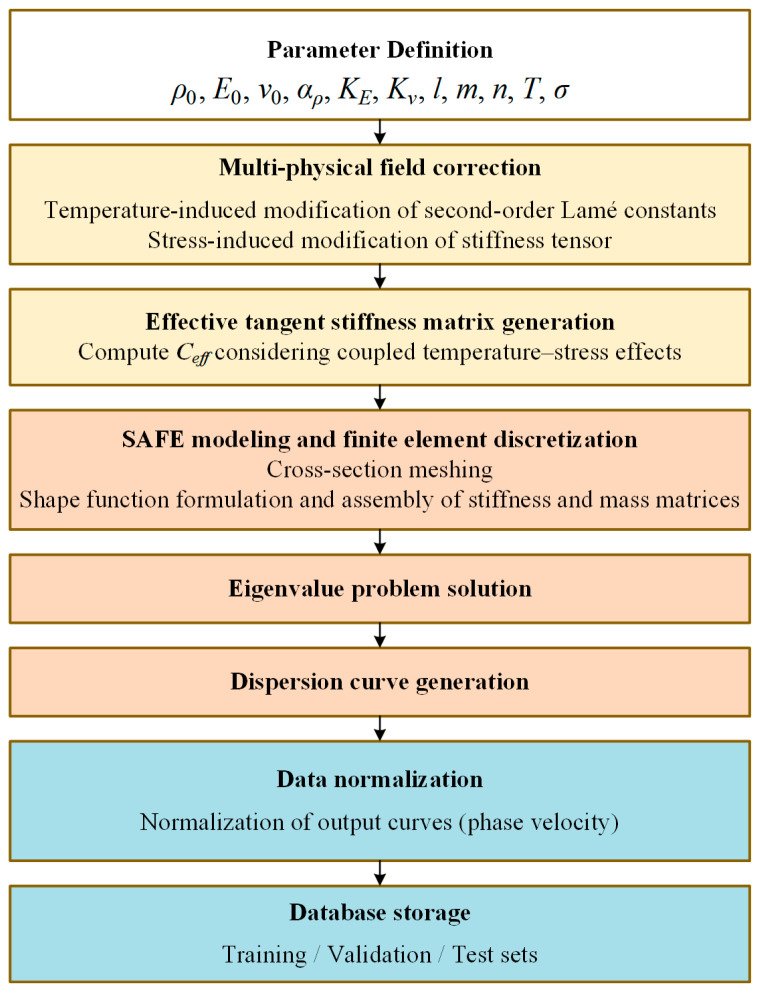
Flowchart of the dispersion curve dataset construction based on SAFE.

Figure 1 illustrates the numerical modeling and dataset generation process for guided-wave dispersion under coupled thermo-mechanical effects. Eleven key physical parameters are first defined as model inputs. The process involves multi-physical field correction of the Lamé constants and stiffness tensor, computation of the effective tangent stiffness matrix considering temperature–stress coupling, and numerical solution using the SAFE method with cross-section meshing and assembly of stiffness and mass matrices. The resulting eigenvalue problem is solved to obtain the dispersion characteristics, followed by curve generation across the designated frequency-thickness range. Finally, the output dispersion data are normalized to ensure numerical stability during training, while the input physical parameters are maintained in their raw physical ranges to form a structured database. This non-normalized input strategy supports the model’s ability to generalize to unseen material configurations.

#### 2.2.2. Dispersion Multi-Head Attention Network

**Figure 2:**
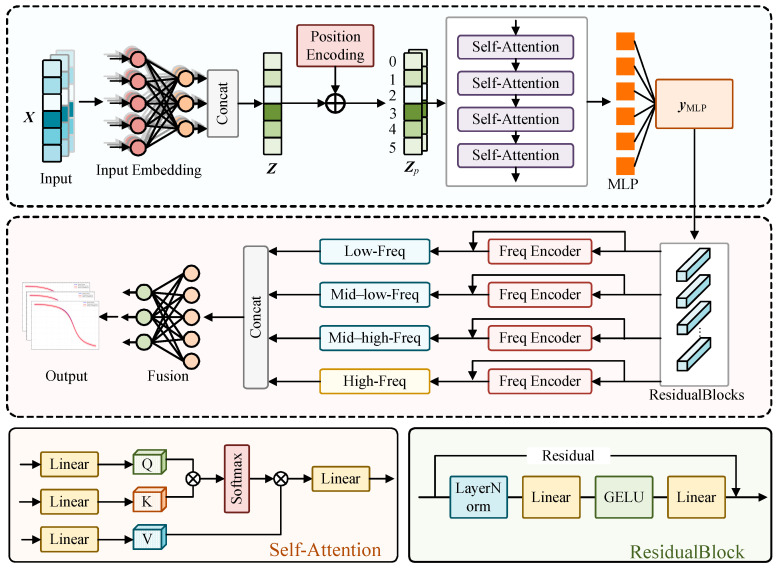
Architecture of the D-MHAN model.

A Dispersion Multi-Head Attention Network (D-MHAN) was developed to rapidly and stably predict phase velocity dispersion curves under extreme temperature–stress conditions. The architecture of the proposed D-MHAN is illustrated in Figure 2. The core concept of this framework is to establish a nonlinear mapping between the input physical parameters and the guided-wave dispersion responses:

(22) $$f(X)\to y,X=[\rho_{0},E_{0},\nu_{0},\alpha_{\rho },K_{E},K_{\nu },l,m,n,T,\sigma ]$$

where *y* denotes the target dispersion curve, and *X* represents the input parameter vector.

To capture the complex and nonlinear coupling among multiple parameters, each input variable is first embedded independently through two fully connected (FC) layers, producing the embedding matrix **Z**:

(23) $$Z=LayerNorm(Gelu(W_{2}\cdot Gelu(W_{1}\cdot X+b_{1})+b_{2}))$$

where ***W***_1_, ***W***_2,_ and ***b***_1_, ***b***_2_ denote the weight matrices and bias vectors of the two FC layers, Gelu is the activation function, and LayerNorm represents layer normalization. A positional encoding term ***P*** is added to the embedding matrix to preserve the positional information of different physical parameters:

(24) $$Z_{P}=Z+P$$

The position-encoded feature matrix is subsequently passed through four Multi-Head Attention (MHA) layers to model the deep coupling mechanism among parameters [35]. Within each attention block, the input feature matrix ***Z****_P_* is projected into three learnable subspaces through linear transformations to obtain the Query (*Q*), Key (*K*), and Value (*V*) matrices:

(25) $$\left\{\begin{array}{c} Q=Z_{P}W^{Q}\\ K=Z_{P}W^{K}\\ V=Z_{P}W^{V} \end{array}\right.$$

The attention score is computed by the scaled dot product of *Q* and *K*, normalized via the Softmax function, and then applied to *V* as a weighted sum:

(26) $$Attention(Q,K,V)=softmax\left(\frac{QK^{T}}{\sqrt{d_{k}}}\right)V$$

where ***d****_k_* is the dimension of the key vector. When the number of attention heads is *m*, this process is executed in parallel *m* times, each with independent linear transformations, thereby enabling the model to learn inter-parameter dependencies within multiple representation subspaces:

(27) $${Head}_{i}=Attention(Z_{P}W_{i}^{Q},Z_{P}W_{i}^{K},Z_{P}W_{i}^{V})$$

All attention-head outputs are concatenated and fused through a final linear layer, yielding the output of the multi-head attention module:

(28) $$MHA(Z_{P})=Concat({Head}_{1},\ldots ,{Head}_{h})W_{o}$$

where ***W****_o_* represents the output projection matrix. After passing through four stacked attention modules with residual connections and normalization, the resulting feature tensor is fed into the final feature-fusion module:

(29) $$y_{MLP}=MLP(MHA(Z_{P}))$$

where the Multilayer Perceptron (MLP), consisting of two FC layers, decodes the learned attention features and regresses them to the target output dimension.

A multi-scale frequency encoder is added after the MLP to improve mapping across different frequency bands. The full-band dispersion response is decomposed into four sub-bands—low, mid-low, mid-high, and high frequencies—each handled by an independent regression sub-network. The predicted results from all sub-bands are concatenated to reconstruct the complete dispersion curve.

The model is trained by minimizing the difference between the predicted and reference curves using the Mean Squared Error (MSE) loss function. This choice ensures uniform precision across the investigated frequency-thickness regime and, combined with a high-density sampling strategy, allows the model to capture both global physical trends and local variations without introducing manual weighting biases.

(30) $$L=MSE=\frac{1}{N}\sum_{j=1}^{N}\;({\hat{y}}_{j}-y_{j}{)}^{2}$$

where y denotes the reference dispersion curve computed via the SAFE method, $\overset{ˆ}{y}$ represents the predicted result, *N* is the number of discrete frequency points along the dispersion curve, and $y_{j}$ and ${\hat{y}}_{j}$ are the true and predicted phase velocities at the *j*-th frequency point. This loss function penalizes the squared deviation between prediction and ground truth, guiding the optimization toward higher prediction accuracy.

The specific configurations of the D-MHAN are summarized in Table 2. The model maps 11 input features to 1001 dispersion points. We utilized the Multi-Head Attention mechanism to decouple complex interactions between material constants and environmental variables. Additionally, four parallel frequency encoders are employed to adapt to distinct dispersion gradients at different frequencies, ensuring high accuracy across the entire spectrum.

**Figure 3:**
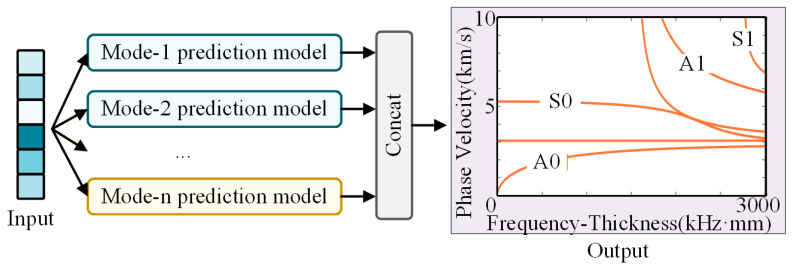
Framework of the multi-modal dispersion curve prediction model.

By integrating the trained sub-networks corresponding to different dispersion modes, the D-MHAN framework enables fast and accurate prediction of multi-modal dispersion curves, as illustrated in Figure 3.

#### 2.2.3. Model Evaluation and Performance Assessment

The D-MHAN’s accuracy and efficiency were evaluated by cross-validation against two numerical methods: the SAFE and Wave Finite Element Method (WFEM). The evaluation procedure covered two scenarios: one without temperature–stress coupling and another with temperature–stress coupling. Furthermore, comparative analyses with different network architectures were conducted to demonstrate the structural advantages and performance gains of the D-MHAN.

Initially, under the condition without temperature–stress coupling, three representative metallic plates—aluminum, steel, and copper—were selected as benchmark materials. Their physical properties are summarized in Table 3. The reference temperature and stress were set to 20 °C and 0 MPa.

Under baseline conditions, S0 and A0 Lamb-wave dispersion curves for each material were computed using SAFE, WFEM, and D-MHAN. Prediction accuracy was quantified using three metrics: Mean Squared Error (MSE), Mean Absolute Error (MAE), and Pearson Correlation Coefficient (PCC).

(31) $$\begin{array}{c} MSE=\frac{1}{N}\sum_{j=1}^{N}\;({\hat{y}}_{j}-y_{j}{)}^{2} \end{array}$$

(32) $$\begin{array}{c} MAE=\frac{1}{N}\sum_{j=1}^{N}\;|{\hat{y}}_{j}-y_{j}| \end{array}$$

(33) $$\begin{array}{c} PCC=\frac{\sum_{j=1}^{N}\;\;({\hat{y}}_{j}-\bar{\hat{y}})(y_{j}-\bar{y})}{\sqrt{\sum_{j=1}^{N}\;\;({\hat{y}}_{j}-\bar{\hat{y}}{)}^{2}\sum_{j=1}^{N}\;\;(y_{j}-\bar{y}{)}^{2}}} \end{array}$$

These metrics, respectively, measure the global fitting precision, the average deviation magnitude, and the correlation consistency between the predicted and reference curves.

Building upon the baseline validation, further experiments were conducted to evaluate the influence of temperature–stress coupling on model performance. In this case, an aluminum plate was used as the study object. Its thermophysical and acoustoelastic parameters are listed in Table 4.

A uniform sampling strategy was applied over the temperature–stress parameter space to generate multiple thermo-mechanical combinations. For each condition, dispersion curves were computed using the SAFE, WFEM, and D-MHAN methods. The computation time of all three approaches was recorded on the same hardware platform, enabling a direct comparison of efficiency while maintaining equivalent accuracy levels.

To verify the effectiveness of the D-MHAN architectural design, additional comparisons were performed against an FCNN model. The predictive results of both models were quantitatively analyzed using the same MSE, MAE, and PCC metrics. The comparative results demonstrate the superior accuracy, stability, and computational efficiency of the proposed D-MHAN framework over traditional numerical solvers and conventional neural architectures.

## 3. Results

### 3.1. Model Training and Performance Evaluation

During the training phase, a total of 4000 parameter combinations were generated through random sampling within the ranges specified in Table 1, based on representative metallic plate materials. For each parameter set, the corresponding ultrasonic guided-wave dispersion curve was obtained using an in-house SAFE solver implemented in MATLAB R2022a and used as the supervised learning target. The dataset was divided into training, validation, and testing subsets in a ratio of 8:1:1. The Adam optimizer was adopted for parameter optimization, and the Mean Squared Error (MSE) was selected as the loss function. To further evaluate the effectiveness of the D-MHAN architectural design, an FCNN was implemented as a comparative baseline model [29].

**Figure 4:**
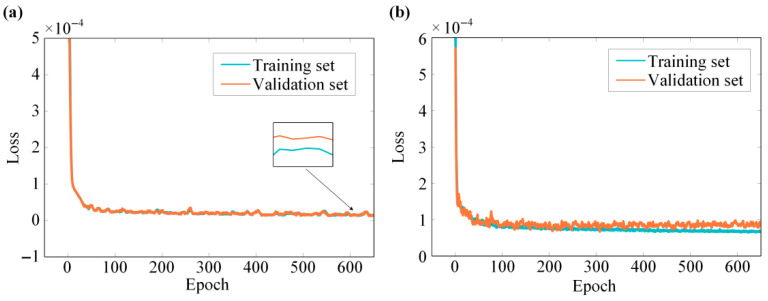
Training loss curves of (**a**) the D-MHAN model and (**b**) the FCNN model.

The training loss evolution of both models is presented in Figure 4. The D-MHAN and FCNN models exhibit stable convergence behavior, with the loss values of both the training and validation sets becoming stable after approximately 600 epochs. Compared with the FCNN, the D-MHAN shows smaller discrepancies between the training and validation loss curves and achieves a lower final steady-state loss value, indicating the absence of overfitting and a more robust generalization capability.

**Figure 5:**
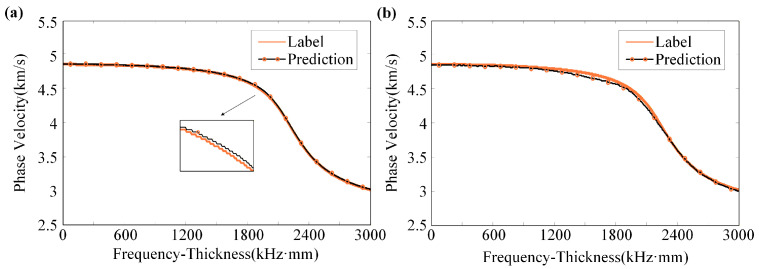
Prediction results for a test sample using (**a**) the D-MHAN model and (**b**) the FCNN model.

To visually assess the model’s generalization capability, a sample was randomly selected from the test dataset for the prediction of the S0-mode dispersion curve, as illustrated in Figure 5a. The D-MHAN prediction exhibits nearly perfect agreement with the reference solution across the entire frequency range, showing no noticeable distortion in either the low- or high-frequency regions, and maintaining fidelity even in the mid-frequency range where the curve shape changes rapidly. In contrast, as shown in Figure 5b, the FCNN model captures the overall trend correctly but displays observable deviations in the mid-frequency region.

**Figure 6:**
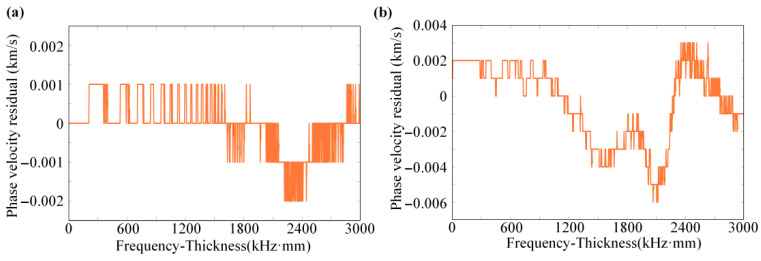
Prediction residuals for a test sample using (**a**) the D-MHAN model and (**b**) the FCNN model.

The corresponding prediction residuals of both models are presented in Figure 6. The D-MHAN exhibits highly stable and accurate predictions, with phase-velocity residuals constrained within ±0.002 km/s. In contrast, the FCNN model shows noticeably larger residuals, particularly in the mid-frequency band, where the maximum error approaches 0.006 km/s.

For an overall quantitative assessment, the three evaluation metrics—MSE, Mean Absolute Error (MAE), and Pearson Correlation Coefficient (PCC)—were calculated across the entire test set. The results, summarized in Table 5, show that the D-MHAN significantly outperforms the FCNN in all aspects. The D-MHAN achieves an MSE of 1.0 × 10^−6^, an MAE of 0.0006, and a PCC of 0.99994, confirming its superior generalization ability and high prediction accuracy.

### 3.2. Dispersion Prediction for Typical Materials

**Figure 7:**
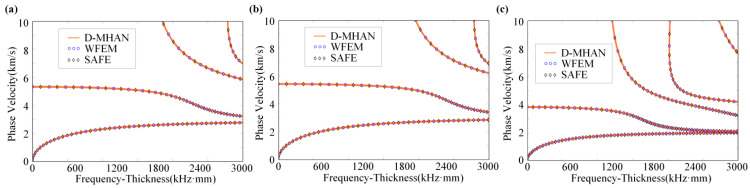
Predicted dispersion curves for (**a**) aluminum, (**b**) steel, and (**c**) copper plates.

To evaluate the model’s generalization capability across different material parameters, three representative engineering materials—aluminum, steel, and copper—were selected under conditions without temperature–stress coupling. The predicted dispersion curves obtained from the D-MHAN were compared with the numerical solutions from the SAFE and WFEMs. The results, shown in Figure 7, demonstrate an excellent overall agreement between the D-MHAN predictions and both numerical references over the entire frequency range.

For all materials, the D-MHAN accurately reproduces the shapes, trends, and magnitudes of both low-order modes (S0, A0) and higher-order modes (S1, A1), confirming that the model captures the intrinsic mapping between material properties and dispersion characteristics rather than overfitting to specific training samples. This finding underscores the D-MHAN’s robustness in delivering reliable predictions across the material parameter space.

For quantitative evaluation, Table 6 lists the performance metrics of the D-MHAN model for the three materials. Despite their substantial differences in mechanical properties, the D-MHAN achieved high prediction accuracy in all cases, with PCC values exceeding 0.9997 and consistently low MSE and MAE values. These results further confirm the model’s excellent generalization ability and its applicability to diverse material systems.

### 3.3. Dispersion Prediction Under Temperature–Stress Coupling

To assess the performance of the D-MHAN model under extreme environments, the dispersion behavior of aluminum plates was predicted within a temperature range of −150 °C to 100 °C and a stress range of 0–100 MPa. The model’s predictive accuracy and computational efficiency were evaluated under these conditions.

**Figure 8:**
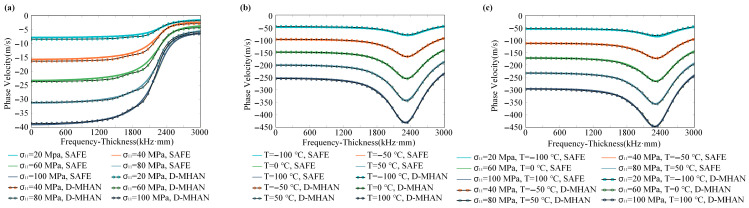
Predicted phase velocity changes in the S0 mode in an aluminum plate under (**a**) different stresses, (**b**) different temperatures, and (**c**) coupled temperature–stress conditions.

Figure 8 compares the predicted phase velocity variations of the S0 mode under three scenarios—(a) stress-only, (b) temperature-only, and (c) coupled temperature–stress conditions—with the numerical reference solutions obtained from the SAFE method. For the stress-only condition, the baseline phase velocity corresponds to 0 MPa; for the temperature-only case, the baseline is −150 °C; and for the coupled case, the baseline is set at −150 °C and 0 MPa. The results indicate that the D-MHAN predictions are in excellent agreement with the SAFE computations. The model accurately captures the dual sensitivity of the S0 mode to both temperature and stress. The temperature effect is more pronounced, leading to a decrease in phase velocity of up to approximately 450 m/s in the mid-high frequency range, with a nonmonotonic dependence on frequency. In contrast, the stress effect primarily manifests as a moderate phase velocity reduction of around 40 m/s in the low-mid frequency range.

**Figure 9:**
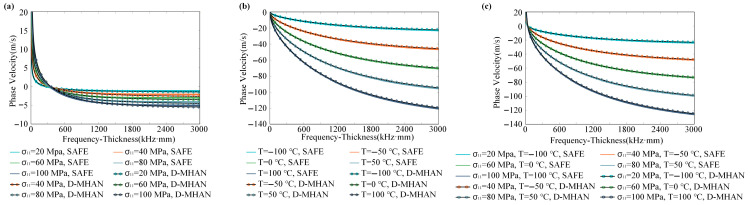
Predicted phase velocity changes in the A0 mode in an aluminum plate under (**a**) different stresses, (**b**) different temperatures, and (**c**) coupled temperature–stress conditions.

Figure 9 presents similar comparisons for the A0 mode. The D-MHAN precisely reproduces the numerical dispersion characteristics obtained from the SAFE method, while revealing the distinctly different physical response behavior of the A0 mode. The results show that the A0 mode is sensitive to temperature but largely insensitive to stress. The phase velocity variation due to temperature increases monotonically with frequency, whereas the stress-induced variation remains negligible.

A comparison between Figure 8a and Figure 9a reveals that the S0 mode exhibits significantly higher sensitivity to stress variations, with a maximum phase velocity deviation of about 40 m/s, while the A0 mode exhibits a much smaller deviation, remaining below 5 m/s. In contrast, the A0 mode yields smaller residuals during dispersion prediction, indicating that its smoother curve shape facilitates more accurate learning and prediction by the model. Comparing Figure 8b and Figure 9b further demonstrates that temperature has a dominant influence on guided-wave dispersion: the temperature-induced phase velocity shifts reach approximately 450 m/s for the S0 mode and 140 m/s for the A0 mode, both substantially larger than the stress-induced effects. Consequently, accurate temperature compensation is essential for ensuring measurement precision in practical monitoring scenarios.

Moreover, as shown in Figure 8c and Figure 9c, the total phase velocity shift under coupled temperature–stress conditions is not a simple linear superposition of the two individual effects but exhibits a distinct nonlinear coupling relationship. The D-MHAN successfully learns and reproduces this nonlinear behavior, demonstrating its capability to model complex multi-physics interactions.

**Figure 10:**
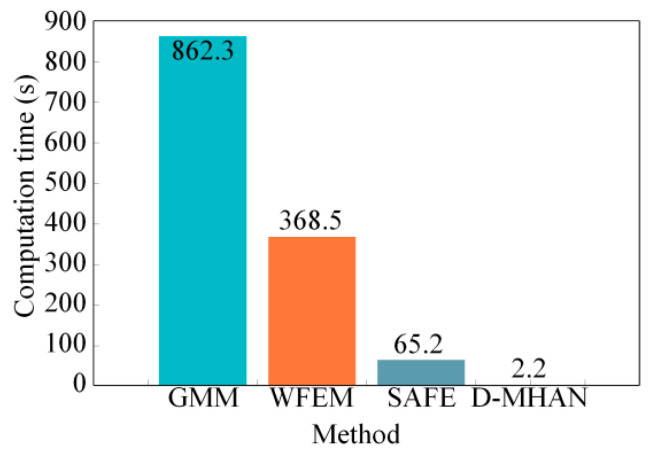
Comparison of computational time for different methods.

Figure 10 compares the computational time required by the D-MHAN, SAFE, and WFEMs on the same hardware platform. It should be noted that the D-MHAN involves a one-time offline development cost of approximately 80 h, which encompasses establishing the high-dimensional parameter space, generating 4000 SAFE datasets, and performing the network training. However, once this initial investment is complete, the D-MHAN enables near-instantaneous analysis. As shown in Figure 10, the D-MHAN completes a full dispersion prediction within 2.2 s, achieving computational speeds approximately 30 and 168 times faster than SAFE and WFEM, respectively. This millisecond-level response is of great significance for real-time structural health monitoring in aerospace applications, where high-fidelity numerical solvers are computationally prohibitive during critical mission windows.

These results validate the D-MHAN’s high-fidelity accuracy relative to numerical methods and superior efficiency in predicting dispersion curves across a high-dimensional parameter space. Moreover, its ability to accurately capture the coupled effects of thermal and mechanical fields provides critical data support for selecting optimal guided-wave modes in various monitoring scenarios—for example, tailoring mode selection based on sensitivity to temperature or stress. The effective combination of high accuracy and fast computation not only enables real-time forward dispersion prediction but also facilitates dynamic compensation for thermo-mechanical effects, significantly enhancing the robustness and reliability of SHM systems under extreme environmental conditions.

Furthermore, it is essential to recognize that while the current D-MHAN framework is validated for uniaxial stress states, the architecture is inherently scalable. As with any mathematical analysis, our model operates within a defined physical scope to ensure high-fidelity mapping. Should applications involve more complex stress orientations or multi-axial loading, the D-MHAN can be systematically extended by expanding the input feature vector to incorporate additional mechanical descriptors. This flexibility, combined with the paradigm shift from iterative numerical solving to instantaneous inference, ensures that the model remains a robust and extensible tool for real-time sensor calibration in extreme environments where traditional solvers face significant computational bottlenecks.

## 4. Discussion

From the preceding results, it is evident that the two representative Lamb modes exhibit markedly different response behaviors under thermo-mechanical coupling. To further elucidate the influence of each input parameter on guided-wave dispersion characteristics, a systematic sensitivity analysis was conducted using the trained D-MHAN model. A hybrid approach combining single-variable perturbation and gradient-based sensitivity evaluation was employed. In this method, one physical parameter was perturbed while keeping all others fixed at their baseline values, thereby isolating its individual contribution to the dispersion curve. Simultaneously, the gradient norm of the model output with respect to each input variable was used to construct a quantitative importance index, and the expectation across multiple samples was taken to ensure stable parameter ranking.

**Figure 11:**
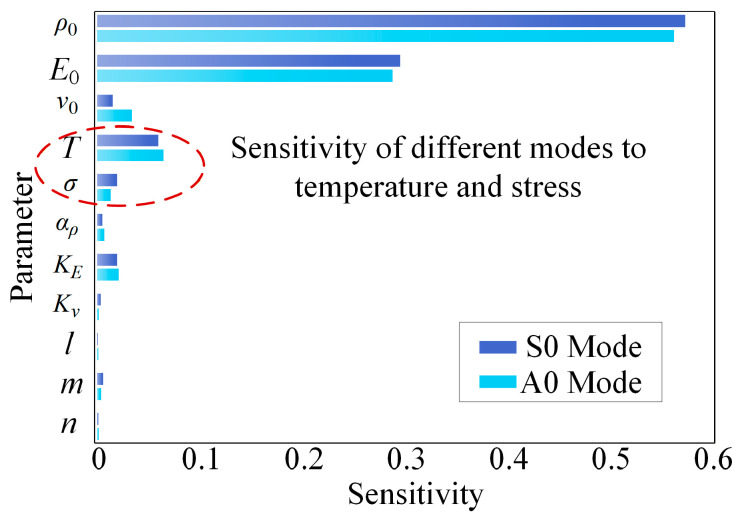
Sensitivity analysis of input parameters on S0 and A0 mode dispersion curves.

The results of this analysis are presented in Figure 11. It can be observed that the fundamental physical parameters—density, Young’s modulus, and Poisson’s ratio—dominate the overall shape of the dispersion curves, consistent with classical wave propagation theory. Among the multi-physics parameters, temperature exhibits the most pronounced effect, corroborating the earlier observation that temperature-induced changes in dispersion are substantially greater than those induced by stress. Consequently, in extreme thermo-mechanical environments, temperature-driven dispersion drift becomes the primary source of uncertainty in guided-wave monitoring, making temperature compensation a crucial component of accurate structural health assessment.

The results in Figure 11 also reveal that the S0 mode exhibits stress sensitivity approximately an order of magnitude higher than that of the A0 mode, whereas the A0 mode is nearly insensitive to stress. This distinct difference in modal response provides a valuable theoretical basis for optimizing monitoring strategies. Specifically, the stress-sensitive S0 mode is more suitable for real-time evaluation of structural load and stress states. During damage monitoring under variable loading conditions, the weak stress sensitivity of the A0 mode can effectively mitigate interference caused by load fluctuations, thereby enhancing robustness and signal-to-noise ratio in damage detection. In thermo-mechanically coupled environments, a hybrid monitoring strategy combining both A0 and S0 modes can be adopted to achieve decoupled assessment of temperature and stress effects.

**Figure 12:**
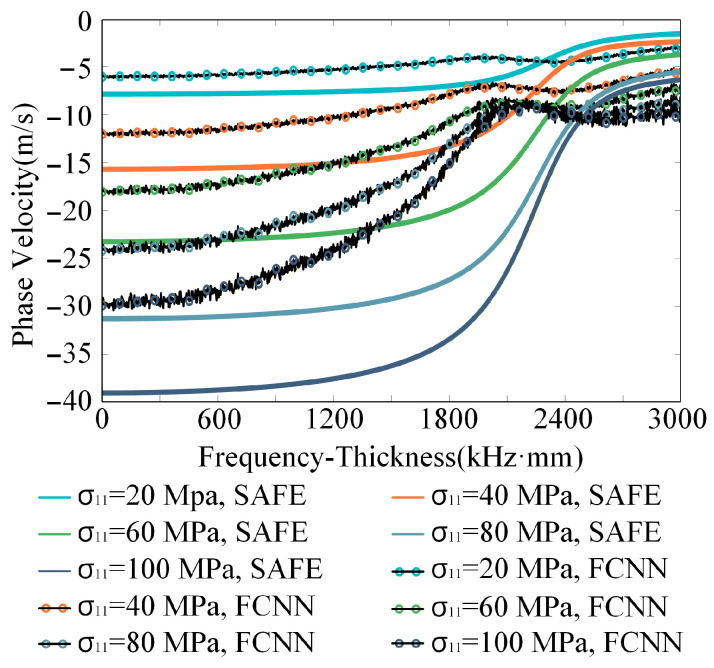
Predicted phase velocity change in S0 mode in an aluminum plate under various stresses using FCNN.

As illustrated in Figure 5, both the D-MHAN and FCNN models produce accurate predictions for the test dataset. However, although the FCNN can roughly capture the overall dispersion trend, its accuracy deteriorates significantly when predicting the phase velocity shifts induced by coupled multi-physical effects, leading to noticeable physical distortion, as shown in Figure 12.

This discrepancy arises from the intrinsic architectural limitations of the FCNN. Its fully connected, flattened structure struggles to learn and disentangle the conditionally dependent and locally coupled relationships inherent in the underlying physical phenomena. In contrast, the MHA mechanism in D-MHAN naturally aligns with the physical reality of such interactions. Through its self-attention operations, the network dynamically evaluates and reinforces the correlations among different physical parameters, thereby achieving functional decoupling of distinct coupling mechanisms. This demonstrates that D-MHAN attains not merely numerical fitting accuracy but also high-fidelity reproduction of physical laws. Such precise characterization of subtle physical shifts is an essential prerequisite for applying the model to environmental effect compensation in practical monitoring systems.

**Figure 13:**
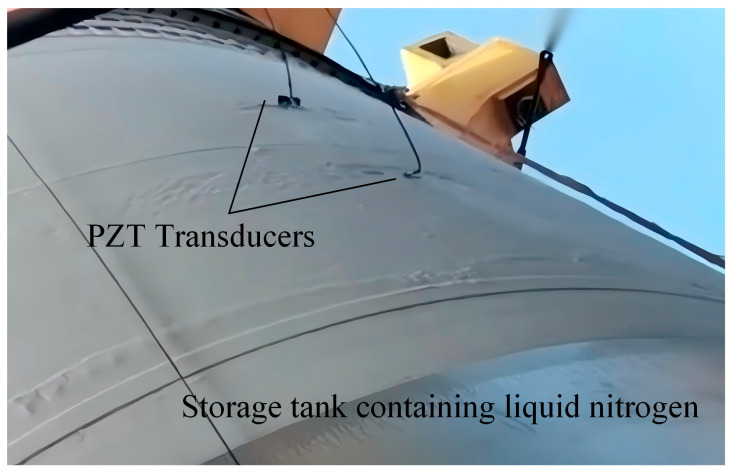
On-site experimental setup for ultrasonic guided wave monitoring of the aerospace storage tank under cryogenic conditions.

**Figure 14:**
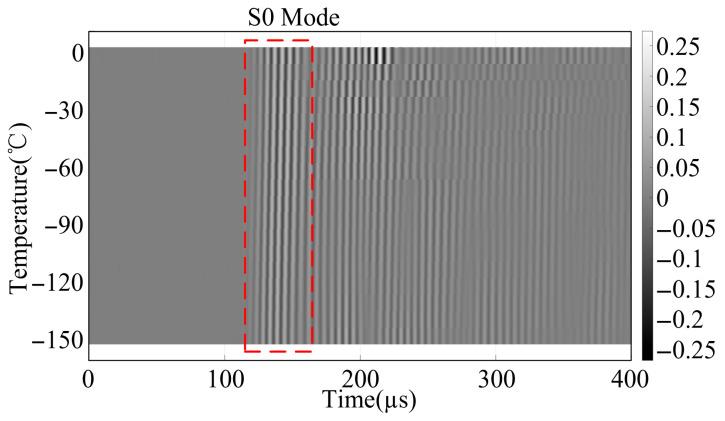
Time-domain response waveforms of the S0 mode excited at 300 kHz evolving with decreasing cryogenic temperatures.

To validate the practical applicability of the proposed model in real-world engineering scenarios, ultrasonic guided wave experiments were conducted on an aerospace cryogenic storage tank. The structure is composed of a 3 mm thick aluminum alloy skin reinforced with stiffeners. Due to the complex boundary reflections and mode conversions induced by these stiffeners, the fundamental symmetric (S0) mode was selected as the primary monitoring signal. This selection relies on the S0 mode’s higher group velocity and lower dispersion characteristics, which facilitate the temporal separation of the direct wave packet from the interference echoes caused by the structural ribs. The piezoelectric sensors were installed with a spacing distance of 0.7 m. A central excitation frequency of 300 kHz was employed to monitor the phase velocity of the first arrival wave under a zero-stress condition across a cryogenic temperature gradient. The experimental setup and the acquired guided wave response signals are illustrated in Figure 13 and Figure 14, respectively. A distinct correlation between temperature reduction and phase velocity shift is observable. Specifically, as the temperature decreased from 0 °C to cryogenic levels, a phase velocity increment of approximately 48 m/s was observed for every 50 °C decrement. This experimental trajectory aligns remarkably well with the predictive results shown in Figure 8, further corroborating the high-fidelity predictive capability of the D-MHAN.

Another important design feature of D-MHAN lies in its non-normalized parameter encoding strategy. Unlike conventional neural networks that normalize input parameters based on training data extrema, D-MHAN directly processes physical quantities in their raw ranges. This enables the model to handle inputs that extend beyond the range of the training dataset. In the material validation experiments for aluminum, steel, and copper, for instance, the density and Young’s modulus of copper exceeded the limits defined in Table 1. Nevertheless, the D-MHAN accurately predicted multi-modal dispersion curves that were highly consistent with the SAFE and WFEM reference results.

This capability confirms that the D-MHAN does not rely on memorizing the training data but instead learns the intrinsic physical mapping between input parameters and dispersion behavior through its attention-driven mechanism. Consequently, the model demonstrates strong generalization and reliable parameter extrapolation capabilities.

For SHM tasks conducted in extreme service environments, such extrapolation capacity is of critical importance. It ensures that the model can still produce physically consistent and accurate predictions when exposed to unseen or out-of-distribution conditions, thereby significantly enhancing both the effectiveness and robustness of the proposed method in real-world engineering applications.

## 5. Conclusions

This study developed a deep learning framework, D-MHAN, to enhance ultrasonic sensor reliability by rapidly predicting guided-wave dispersion curves under coupled extreme temperature–stress conditions. The proposed model accepts eleven geometry–material–environment parameters as inputs and achieves end-to-end prediction through independent embedding, multi-head attention interaction, and residual feature fusion.

By adopting a non-normalized input strategy to internalize underlying physical laws, the model demonstrates superior robustness in high-dimensional physical mapping, ensuring physically consistent predictions even for material configurations that fall outside the training manifold. Validated against a high-fidelity database generated by the SAFE method, the D-MHAN achieves a Pearson correlation coefficient of 0.9999 and maintains high accuracy across the entire frequency-thickness regime. Comparative studies on aluminum, steel, and copper plates confirm the model’s robustness across different materials.

In terms of computational efficiency, although the framework involves a one-time offline development cost of approximately 80 h for dataset generation and training, it enables a paradigm shift to near-instantaneous inference. The D-MHAN completes a full dispersion prediction in 2.2 s, achieving computational speeds approximately 30 and 168 times faster than SAFE and WFEM. The model’s practical utility is further corroborated by cryogenic experiments on an aerospace storage tank, where the predicted phase-velocity shifts exhibited high consistency with measured data under extreme thermal fields.

Overall, the proposed approach provides an efficient and reliable solution for real-time sensor signal interpretation and environmental effect compensation in extreme service environments. The architecture is inherently scalable, allowing for systematic extension to multi-axial loading or complex waveguides by expanding the input feature vector without altering the core logic. This work offers a valuable technical foundation for deploying intelligent ultrasonic sensors in aerospace, deep-sea exploration, and energy systems, where real-time and high-fidelity modeling of wave propagation is critical for ensuring operational safety.

## Figures and Tables

**Table 1:** Range of input parameters for the training dataset.

Material Property	Symbol	Range	Unit
Density	*ρ*_0_	2000~6000	kg/m^3^
Young’s modulus	*E*_0_	60~80	GPa
Poisson’s ratio	*ν*_0_	0.2~0.4	/
Third-order Murnaghan constant	*l*	−100~−600	GPa
	*m*	−200~−600	GPa
	*n*	−250~−600	GPa
Linear thermal expansion coefficient	$\alpha_{L}$	0.000006~0.00003	1/°C
Temperature coefficient of Young’s modulus	$K_{E}$	−0.0006~−0.0002	GPa/°C
Temperature coefficient of Poisson’s ratio	$K_{\nu }$	−0.00002~0	1/°C
Temperature	*T*	−250~100	°C
Stress	σ	0~150	MPa

**Table 2:** Hyperparameters and structural configurations of the D-MHAN.

Hyperparameter	Symbol	Value	Description
Hidden Dimension	*d_model_*	576	Latent space dimension
Number of Heads	*m*	12	Parallel attention heads
Head Dimension	*d_k_*, *d_v_*	48	Dimensionality per head
Attention Layers	*N_attn_*	4	Blocks for parameter decoupling
Residual Blocks	*N_res_*	8	Depth of feature extraction
Feature Fusion	-	[1728, 1152, 576]	Neurons in the 3-layer fusion stage
Frequency Encoders	-	4	Multi-scale frequency modeling
Dropout Rate	-	0.08	Regularization for stable training
Activation	-	GELU	Nonlinear activation function

**Table 3:** Physical parameters of typical materials.

Material	Density (kg/m^3^)	Young’s Modulus (GPa)	Poisson’s Ratio
Aluminum	2700	68	0.33
Steel	7850	210	0.3
Copper	8960	117	0.34

**Table 4:** Thermophysical and acoustoelastic parameters of the aluminum plate.

Material Property	Symbol	Range	Unit
Third-order Murnaghan constant	*l*	−252.2	GPa
	*m*	−324.9	GPa
	*n*	−351.2	GPa
Linear thermal expansion coefficient	$\alpha_{L}$	0.0000236	1/°C
Temperature coefficient of Young’s modulus	$K_{E}$	−0.000458	GPa/°C
Temperature coefficient of Poisson’s ratio	$K_{\nu }$	0	1/°C

**Table 5:** Performance comparison of D-MHAN and FCNN models on the test set.

Model	MSE	MAE	PCC
D-MHAN	1.0 × 10^−6^	0.0006	0.99994
FCNN	1.6 × 10^−5^	0.0029	0.99945

**Table 6:** Prediction performance metrics of the D-MHAN model on different materials.

Material	MSE	MAE	PCC
Aluminum	1.5 × 10^−7^	0.0002	0.99996
Steel	0.9 × 10^−6^	0.0008	0.99983
Copper	1.1 × 10^−6^	0.0007	0.99979

## Data Availability

The original contributions presented in this study are included in the article. Further inquiries can be directed to the corresponding author.
